# Oral administration of *Porphyromonas gingivalis* to mice with diet-induced obesity impairs cognitive function associated with microglial activation in the brain

**DOI:** 10.1080/20002297.2024.2419155

**Published:** 2024-11-14

**Authors:** Kana Oue, Yosuke Yamawaki, Kazuhisa Ouhara, Eiji Imado, Tetsuya Tamura, Mitsuru Doi, Yoshitaka Shimizu, Mitsuhiro Yoshida, Noriyoshi Mizuno, Norimitsu Morioka, Takashi Kanematsu, Masahiro Irifune, Yukio Ago

**Affiliations:** a Department of Dental Anesthesiology, Division of Oral and Maxillofacial Surgery and Oral Medicine, Hiroshima University Hospital, Hiroshima, Japan; b Department of Advanced Pharmacology, Daiichi University of Pharmacy, Fukuoka, Japan; c Department of Periodontal Medicine, Division of Applied Life Sciences, Institute of Biomedical & Health Sciences, Hiroshima University, Hiroshima, Japan; d Department of Dental Anesthesiology, Graduate School of Biomedical and Health Sciences, Hiroshima University, Hiroshima, Japan; e Department of Pharmacology, Graduate School of Biomedical and Health Sciences, Hiroshima University, Hiroshima, Japan; f Department of Cell Biology, Aging Science, and Pharmacology, Faculty of Dental Science, Kyushu University, Fukuoka, Japan; g Department of Cellular and Molecular Pharmacology, Graduate School of Biomedical and Health Sciences, Hiroshima University, Hiroshima, Japan

**Keywords:** Periodontal disease, *Porphyromonas gingivalis*, oral infection, obesity, cognitive dysfunction, inflammation, microglia

## Abstract

**Objective:**

Both periodontal disease and obesity are risk factors for dementia, but their links to 1brain function remain unclear. In this study, we examined the effects of oral infection with a periodontal pathogen on cognitive function in a mouse model of obesity, focusing on the roles of microglia.

**Methods:**

To create a mouse model of diet-induced obesity and periodontitis, male C57BL/6 J mice were first fed a high-fat diet containing 60% lipid calories for 18 weeks, beginning at 12 weeks of age, to achieve diet-induced obesity. Then, *Porphyromonas gingivalis* administration in the oral cavity twice weekly for 6 weeks was performed to induce periodontitis in obese mice.

**Results:**

Obese mice orally exposed to *P. gingivalis* showed cognitive impairment in the novel object recognition test. Increased expression levels of inflammatory cytokines (e.g. interleukin-1β and tumor necrosis factor-α) were observed in the hippocampus of *P. gingivalis*-treated obese mice. Immunohistochemical analysis revealed that microglia cell body size was increased in the hippocampus and prefrontal cortex of *P. gingivalis*-treated obese mice, indicating microglial activation. Furthermore, depletion of microglia by PLX3397, a colony-stimulating factor 1 receptor inhibitor, ameliorated cognitive dysfunction.

**Conclusion:**

These results suggest that microglia mediate periodontal infection-induced cognitive dysfunction in obesity.

## Introduction

Dementia is a syndrome in which cognitive decline exceeds the degree that may be expected as a usual consequence of biological aging. Currently, more than 55 million people worldwide have dementia, and there are nearly 10 million new cases annually (World Health Organization, 2022). Because of increases in population growth and aging, the number of people with dementia is expected to nearly triple to >152 million by 2050 [1]. The increasing prevalence of neurodegenerative diseases such as Alzheimer’s disease (AD) because of rapid population aging has become a global public health problem, but there remains no established cure. Various risk factors are involved in the onset of dementia, including hypertension, midlife obesity, diabetes, physical inactivity, genetics, and aging [2]. Moreover, microbes may contribute to dementia and cognitive impairment. For example, *Chlamydia pneumoniae* [3] and *Borrelia burgdorferi* [4] have been found in the blood and cerebrospinal fluid of patients with AD. Additionally, a case-control study showed that patients with infections were twofold more likely to experience dementia, compared with people who did not have infections [5].

Periodontal disease, a local oral infection with chronic inflammation caused by oral bacteria, has been associated with an increased risk of systemic inflammatory diseases. Periodontopathogenic bacteria and their toxins have been reported to cause systemic chronic inflammation, which is associated with various diseases (e.g. cardiovascular disease and diabetes). Furthermore, epidemiological studies in recent decades have suggested that these bacteria and toxins constitute risk factors for AD [6,7]. *Porphyromonas gingivalis* (i.e. *P. gingivalis*), a representative periodontopathogenic bacterium, is a keystone pathogen of oral inflammation that subverts host immune system and qualitatively alters the oral flora, triggering inflammation and other immune disruptions that contribute to periodontitis, a severe form of periodontal disease [8,9]. Lipopolysaccharide (LPS) derived from *P. gingivalis* (i.e. *P. gingivalis* LPS) has been detected in the brains of patients with AD [10]. Toxic proteases from *P. gingivalis* (known as gingipains) were also identified in the brains of patients with AD; their levels were correlated with the degrees of tau protein and ubiquitin pathology [11]. These findings suggest that periodontal disease is involved in the development and progression of AD [12].

Obesity, a risk factor for dementia, increases the risks of various diseases by causing adipocyte hypertrophy, which increases the production of inflammatory adipokines (i.e. adipocyte-derived bioactive substances) including interleukin (IL)-6, tumor necrosis factor (TNF)-α, monocyte chemoattractant protein-1, and leptin; this process leads to mild chronic inflammation throughout the body [13]. The activation of microglia, immune cells within the brain, is also involved in obesity-associated cognitive dysfunction [14]. Notably, studies in recent decades have provided evidence to support a link between obesity and periodontal disease. For example, *P. gingivalis* LPS causes inflammatory adipokine production and insulin resistance in adipose tissue [15]; moreover, *P. gingivalis* infection enhances inflammation and fibrosis in non-alcoholic fatty liver disease [16] and non-alcoholic steatohepatitis [17]. Although the effects of interactions between obesity and periodontal disease on brain function remain unclear, periodontal infection can cause microglial activation in the cortex and hippocampus [18,19]. These findings imply that periodontal infection in the context of obesity can enhance inflammation both in the periphery and in the brain, thereby exacerbating central nervous system impairments such as cognitive dysfunction.

In this study, we aimed to evaluate the effects of short-term periodontal infection on cognitive function and brain inflammation in a mouse model of obesity. Accordingly, we conducted intraoral administration of the periodontal pathogen *P. gingivalis* to diet-induced obese mice for 6 weeks; we then analyzed behavior, inflammation-related gene expression, and microglial activation in the brain.

## Materials and methods

### Preparation of bacteria

The periodontopathogenic bacteria used in this study were purchased from ATCC. The gram-negative, rod-shaped anaerobe *P. gingivalis* strain W83 was cultured at 37°C on sheep blood agar plates in an anaerobic environment established using the Anaeropack system (Mitsubishi Gas Chemical Company, Tokyo, Japan). After 2-day incubation, *P. gingivalis* was inoculated in liquid medium (Trypticase Soy Broth containing 1% yeast extract with hemin and menadione). Broth cultures of bacteria were harvested during the exponential growth phase, washed with phosphate-buffered saline (PBS), and used for experiments.

### Animals

Male C57BL/6 J mice (The Jackson Laboratory Japan, Yokohama, Japan) were housed in a specific pathogen-free facility at a constant temperature of 22 ± 1°C, with a standard 12-h light/dark cycle (lights on 08:00). The mice were provided *ad libitum* access to a normal laboratory diet (ND; Oriental MF [3.59 kcal/g, 5% fat, 23% protein, 70% carbohydrate], Oriental Yeast Co., Tokyo, Japan) and water. All animal experimental procedures used in this study were approved by the Committee of Research Facilities for Laboratory Animal Science of Hiroshima University (approval No. A20-22) and were conducted in accordance with the Guide for the Care and Use of Laboratory Animals (National Research Council Institute for Laboratory Animal, 1996) and ARRIVE guidelines [20,21].

### Model of diet-induced obesity with periodontitis in middle-aged mice

**Figure 1.**
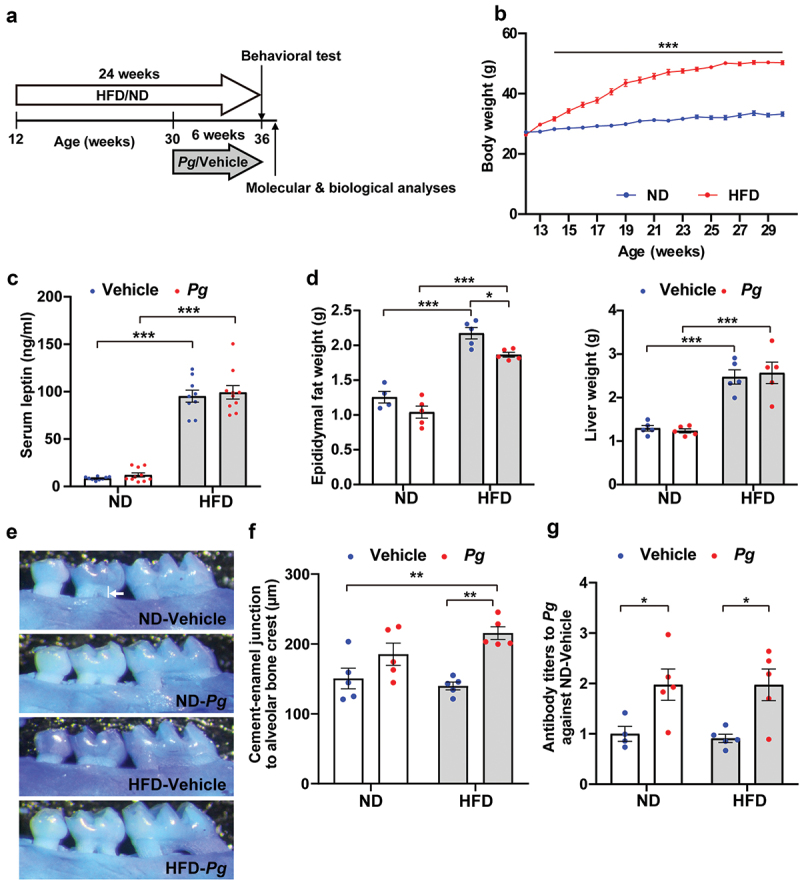
Effects of long-term high-fat diet intake and oral administration of *P. gingivalis* in mice. (a) Timeline of experimental procedures, including administration of respective diets and oral administration of *P. gingivalis* (*Pg*). (b) Body weights of mice fed a normal diet (ND) and high-fat diet (HFD) were measured up to 30 weeks of age. Results are expressed as the mean ±S.E.M. of 10 mice per group. ***P < 0.001. Serum leptin levels (c) and weights of epididymal fat and liver (d) were analyzed in 36-week-old mice. Results are expressed as the mean ±S.E.M. of 10 (c) and five (d) mice per group. *P < 0.05, ***P < 0.001. (e, f) Alveolar bone levels of the upper jaw were analyzed. Representative images are shown (e); the distance from the alveolar bone crest of the proximal buccal root of the second molar to the cementoenamel junction (indicated by white line in e) was measured (f). Results are expressed as the mean ±S.E.M. of five mice per group. **P < 0.01. (g) Serum *Pg* antibody titer was measured by ELISA, and the data were expressed as a ratio to the ND-vehicle group. Results are expressed as the mean ±S.E.M. of five mice per group. **P < 0.01.

A model of diet-induced obesity with periodontitis was created in middle-aged mice, using the following method. Mice were fed a high-fat diet (HFD) containing 60% lipid calories (HFD-60 [4.93 kcal/g, 35% fat, 23% protein, 25% carbohydrate], Oriental Yeast Co., Tokyo, Japan) *ad libitum* for 24 weeks, beginning at 12 weeks of age, to achieve diet-induced obesity. Next, 10^8^ colony-forming units of *P. gingivalis* were suspended in 100 μl of PBS containing 2% carboxymethyl cellulose; the suspension was applied to the molars of each mouse twice weekly for 6 weeks, beginning at 30 weeks of age [22] (Figure 1a). As a control, the vehicle group was applied with 100 μl of PBS containing 2% carboxymethyl cellulose without *P. gingivalis*.

### Depletion of microglia

The colony-stimulating factor 1 (CSF1) receptor is required for microglia development and maintenance; pharmacological inhibition of the CSF1 receptor in adulthood can largely eliminate microglia [23]. Therefore, we used a CSF1 receptor inhibitor PLX3397 to induce depletion of microglia. A robust and time-dependent reduction in brain microglia number, with a 50% reduction in microglia, was observed after just 3 days of treatment with PLX3397 (290 mg/kg chow) [23]. Additionally, the number of microglia in the brain was stably reduced by more than 70% over 1–2 weeks of the PLX3397 treatment. Thus, in this study, mice were treated with PLX3397 for 10 days. PLX3397 was incorporated into the AIN-76A rodent diet (Research Diets, Inc., New Brunswick, NJ, USA) at 290 mg/kg chow and administered to HFD-fed mice with or without *P. gingivalis* infection, beginning at 35 weeks of age. The AIN-76A rodent diet was used as a control treatment. Ten days after the initiation of PLX3397 administration, the novel object recognition test was performed.

### Novel object recognition test

The novel object recognition test assesses memory ability by quantifying object recognition [24,25]. The novel object recognition test was performed as described in our previous report [26]. Briefly, mice were habituated to an experimental box (30 × 30 × 35 cm^3^) under dim light conditions (30 lux), 10 min daily for 3 consecutive days. In the training session, two novel objects (*a* and *b*) were placed in the box, and the mouse was allowed to explore freely for 10 min. In the retention session (conducted 1 h after the training session), object ‘*b*’ was replaced by a novel object ‘*c*’, and the mouse was allowed to explore the same box for 5 min. The time spent exploring each object was measured during the training and retention sessions. Recognition memory was evaluated using the discrimination index, which was defined as the difference between the exploration time for the novel object and the exploration time for the familiar object, divided by the total exploration time. Behavioral analysis was conducted by experimenters who were blinded to the treatment conditions of the mice.

### Evaluation of alveolar bone levels

Mouse alveolar bone levels were evaluated using the method of Kawai et al. [27]. To distinguish the cementoenamel junction, decalcified mouse maxillae were stained with methylene blue (Sigma-Aldrich, St. Louis, MO, USA). The distance from the alveolar bone crest of the proximal buccal root of the second molar to the cementoenamel junction was measured using ImageJ software (National Institutes of Health, Bethesda, MD, USA).

### Measurement of serum leptin levels

Serum samples were collected from each mouse, and leptin concentrations were measured using a Mouse Leptin Assay Kit (IBL, Gunma, Japan), in accordance with the manufacturer’s instructions. Leptin concentrations were determined by reference to a standard curve, which had been prepared by serial dilution. Each sample was examined in triplicate in a 96-well enzyme-linked immunosorbent assay (ELISA) plate.

### Quantitative real-time polymerase chain reaction (qRT-PCR) analysis

Total RNA was extracted from mouse hippocampal tissue using the FastGene RNA Basic Kit (Nippon Genetics Co., Ltd., Tokyo, Japan), in accordance with the manufacturer’s instructions. cDNA synthesis and qRT-PCR were performed as described in our previous report [28]. cDNA was synthesized using the Verso cDNA Synthesis Kit (Thermo Fisher Scientific, Waltham, MA, USA) with the Biometra TProfessional Basic Gradient 96 thermal cycler (Göttingen, Germany). qRT-PCR was performed using THUNDERBIRD Next SYBR qPCR Mix (TOYOBO, Osaka, Japan) on the StepOnePlus Real Time PCR system (Applied Biosystems, Carlsbad, CA, USA). The following PCR protocol was used: DNA polymerase activation at 95°C for 1 min, followed by 40 cycles of denaturation at 95°C for 5 s and annealing/extension at 60°C for 30s. Ct values were normalized to the levels of glyceraldehyde-3-phosphate dehydrogenase (GAPDH), a reference gene that does not undergo adipogenesis-related changes in expression; relative gene expression levels were calculated using the *ΔΔ*CT method. The primer sequences used here were shown in Supplemental table.

### Immunohistochemistry

Ionized calcium-binding adapter molecule 1 (Iba1) immunostaining was performed as described in our previous report [29]. Briefly, mice were transcardially perfused with ice-cold PBS, followed by 4% paraformaldehyde phosphate buffer solution (#09154-85; Nacalai Tesque, Inc., Kyoto, Japan). Then, the whole brain of each mouse was removed and post-fixed in the same fixative. Brains were dehydrated by immersion in 30% sucrose in PBS at 4°C overnight. Twenty-micrometer-thick sections were prepared using a cryostat. Sections were permeabilized with 0.2% Triton X-100 in PBS for 20 min, then blocked using 5% goat serum in PBS with 0.03% Triton X-100 for 1 h at room temperature. Then, they were incubated with a rabbit anti-Iba1 polyclonal antibody (1:500, RRID: AB_839504; WAKO Pure Chemical Industries, Osaka, Japan) at 4°C overnight, followed by an Alexa Fluor 488-labeled anti-rabbit IgG (1:200, RRID: AB_143165; Thermo Fisher Scientific) for 2 h at room temperature. Sections were washed in PBS, mounted on slides, and coverslipped with Prolong Gold Antifade Reagent containing 4’,6-diamidino-2-phenylindole (DAPI) (Thermo Fisher Scientific). Fluorescence images were acquired using a fluorescence microscope (BZ-X800; Keyence, Elmwood Park, NJ, USA). The number of Iba1-positive microglia cells and the cell body area in the left and right hippocampus and prefrontal cortex were counted for each section, and the counts were averaged for each animal. All immunohistochemical experiments and analyses were performed in a blinded manner by experimenters who were unaware of the treatment conditions.

### Statistical analysis

The sample size for experiments was determined by referring to relevant research papers using the same behavioral tests and biochemical/immunohistochemical methods [19,26,30–33]. All data are presented as the mean ± standard error of the mean (S.E.M.). Data were analyzed by Student’s *t*-test for comparisons between two groups or by two-way analysis of variance, followed by the Tukey–Kramer test. Statistical analyses were conducted using the JMP® 16 software package (SAS Institute, Cary, NC, USA). *P*-values < 0.05 were considered statistically significant.

## Results

### Evaluation of diet-induced obesity with periodontitis in middle-aged mice

At 2 weeks after initiation of the HFD, mice fed an HFD (i.e. HFD group) weighed significantly more than mice fed an ND (i.e. ND group) (Figure 1b). At 30 weeks of age, body weight gain was significantly greater in the HFD group (24.0 ± 0.47 g) than in the ND group (6.0 ± 0.63 g). Serum leptin levels in 36-week-old mice were approximately 10-fold higher in the HFD group than in the ND group (Figure 1c). Mice in the HFD group also showed significant increases in epididymal white adipose tissue weight and liver weight (Figure 1d). These findings suggested that mice in the HFD group had become obese. In the HFD group, serum leptin levels and liver weight were not affected by oral administration of *P. gingivalis*, whereas epididymal white adipose tissue weight was rather decreased by periodontal infection (Figures 1c,d).

To assess periodontitis status, we analyzed the distance from the alveolar bone crest of the proximal buccal root of the second molar to the cementoenamel junction (Figures 1e,f), as well as serum *P. gingivalis* antibody titers (Figure 1g), in 36-week-old mice that had undergone intraoral administration of *P. gingivalis* for 6 weeks. Periodontal infection for 6 weeks significantly lowered the alveolar crest position in HFD-fed mice (HFD-*Pg* group), compared with vehicle-treated HFD-fed mice (HFD-vehicle group) and ND-fed mice (ND-vehicle group). There was no significant change in alveolar crest position between *P. gingivalis*-treated ND-fed mice (ND-*Pg* group) and mice in the ND-vehicle group. Additionally, mice in the ND-*Pg* and HFD-*Pg* groups showed increased levels of serum *P. gingivalis* antibody titers, compared with mice in the ND-vehicle and HFD-vehicle groups, respectively.

### Effects of diet-induced obesity and periodontal infection on cognitive function

**Figure 2.**
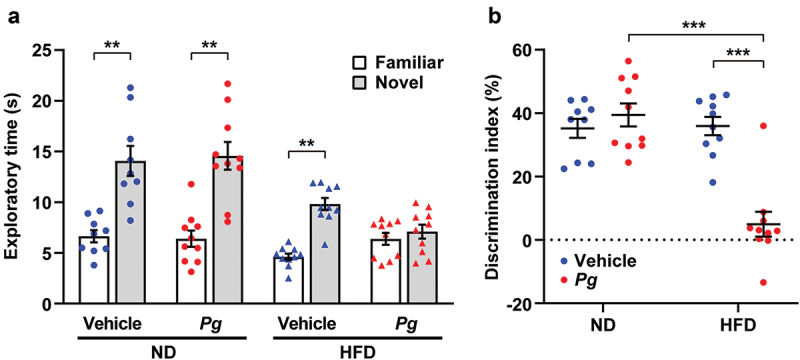
Effects of diet-induced obesity and periodontal infection on cognitive function. Recognition memory was analyzed by the novel object recognition test. Time spent exploring each object (a) and discrimination index (b) are shown. ND; normal diet, HFD; high-fat diet, *Pg; P. gingivalis*. Results are expressed as the mean ± S.E.M. of 10 mice per group. **P < 0.01, ***P < 0.001.

To investigate the effects of periodontal infection and obesity on cognitive function, we subjected the mice to novel object recognition tests (Figure 2). Mice with good object recognition memory were expected to spend more time exploring the novel object than the familiar object during the retention session. Consistent with this expectation, mice in the ND-vehicle group spent more time exploring the novel object than the familiar object (Figure 2a). Mice in the ND-*Pg* and HFD-vehicle groups also spent preferentially more time exploring the novel object. However, mice in the HFD-*Pg* group did not demonstrate a preference for the novel object. Additionally, the discrimination index was significantly lower in the HFD-*Pg* group than in the other groups (Figure 2b). These results suggest that oral infection with *P. gingivalis* in the context of diet-induced obesity causes cognitive dysfunction.

### Inflammatory responses in the hippocampus of *P. gingivalis*-treated obese mice

**Figure 3.**
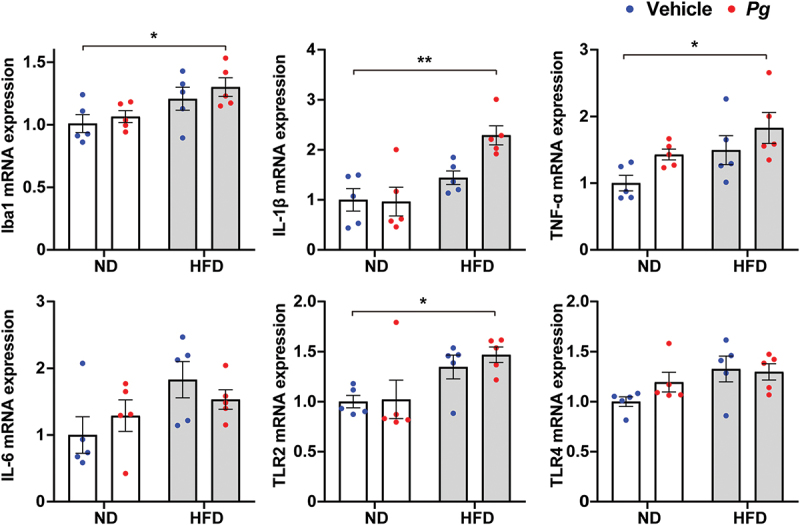
Effects of diet-induced obesity and periodontal infection on inflammation-related gene expression in the mouse hippocampus. mRNA levels were analyzed by quantitative reverse transcription-polymerase chain reaction, and the data were normalized to the level of the housekeeping gene GAPDH. ND; normal diet, HFD; high-fat diet, *Pg; P. gingivalis*. Results are expressed as the mean ±S.E.M. of five mice per group. *P < 0.05, **P < 0.01.

Next, we analyzed the mRNA expression patterns of inflammation-related genes in the hippocampus, a brain region with important roles in learning and memory consolidation [34] (Figure 3). The expression levels of the microglial marker Iba1, the proinflammatory cytokines IL-1β and TNF-α, and the pattern recognition receptor Toll-like receptor 2 (TLR2) were significantly higher in the HFD-*Pg* group than in the ND-vehicle group. It is therefore possible that the increased inflammatory response in the hippocampus is an additive or synergistic effect between periodontal infection and obesity, while there were no significant differences between the HFD-*Pg* group and the ND-*Pg* or HFD-vehicle groups. There were no significant differences among groups in the mRNA expression levels of IL-6 or TLR4.

### Increased microglial proliferation and activation in the brains of *P. gingivalis*-treated obese mice

**Figure 4.**
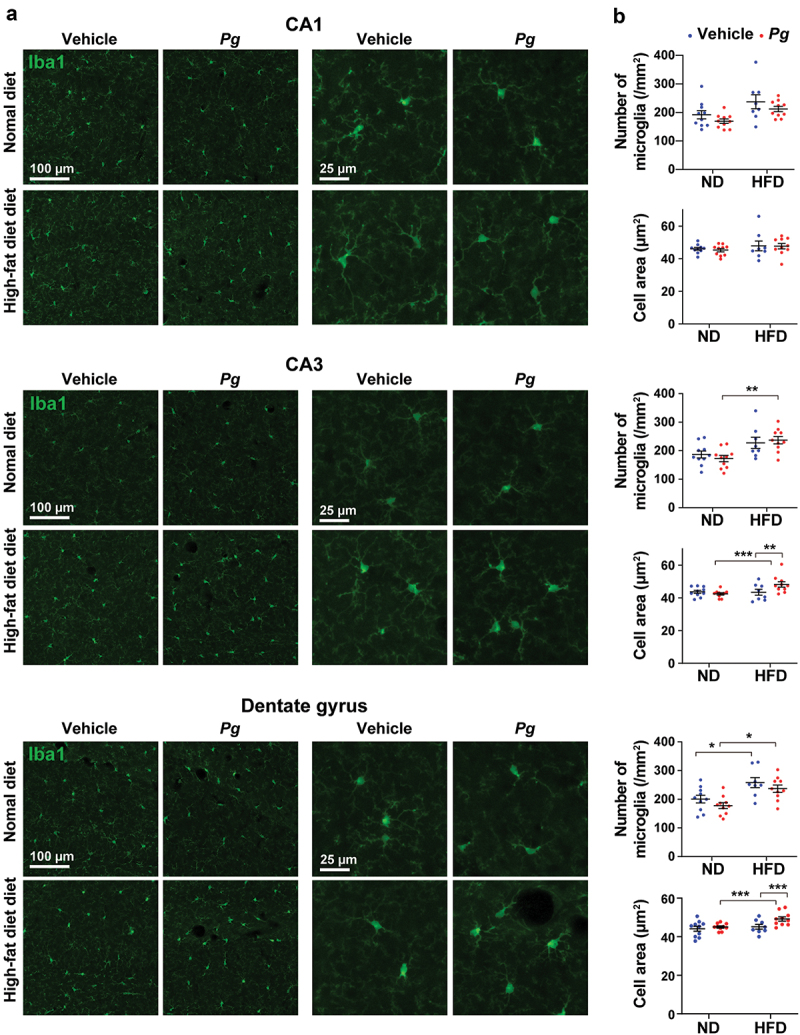
Microglial proliferation and enlargement in the hippocampus of *P. gingivalis*-treated obese mice. (a) Representative images of Iba1-positive cells in CA1, CA3, and dentate gyrus regions of the hippocampus in 36-week-old mice are shown. (b) The number of microglia and mean cell area per Iba1-labeled microglia in each region of the hippocampus are shown. The numbers of microglia were counted in each left and right hemisphere section of each mouse; 10 total sections were analyzed from five mice per group. For analysis of the cell area of microglia, five microglia per left and right hemisphere section were randomly selected from each mouse, and the values were averaged for each section. Ten total sections were analyzed from five mice per group. ND; normal diet, HFD; high-fat diet, *Pg; P. gingivalis*. Results are expressed as the mean ±S.E.M. *P < 0.05, **P < 0.01, ***P < 0.001.

**Figure 5.**
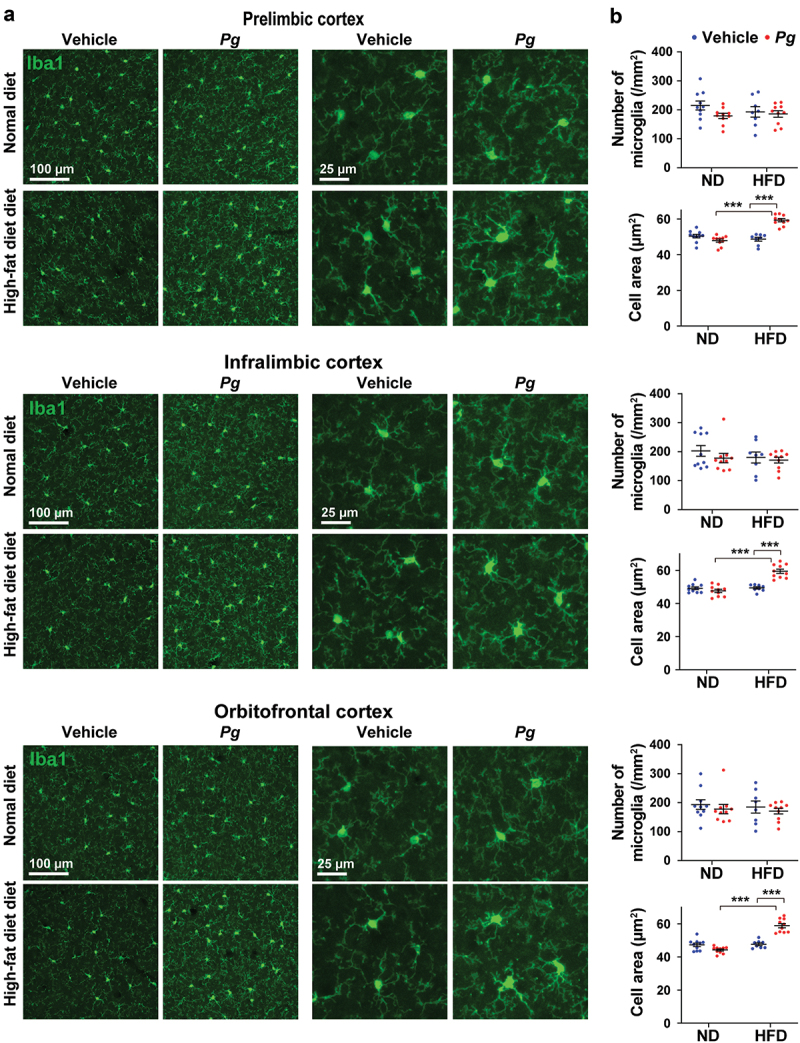
Microglial proliferation and enlargement in the prefrontal cortex of *P. gingivalis*-treated obese mice. (a) Representative images of Iba1-positive cells in the prelimbic, infralimbic, and orbitofrontal cortices in 36-week-old mice are shown. (b) The number of microglia and mean cell area per Iba1-labeled microglia in each region of the hippocampus are shown. The numbers of microglia were counted in each left and right hemisphere section of each mouse; 10 total sections were analyzed from five mice per group. For analysis of the cell area of microglia, five microglia per left and right hemisphere section were randomly selected from each mouse, and the values were averaged for each section. Ten total sections were analyzed from five mice per group. ND; normal diet, HFD; high-fat diet, *Pg; P. gingivalis*. Results are expressed as the mean ±S.E.M. ***P < 0.001.

To identify changes in microglial expression patterns and evaluate microglial morphology in the brain, we performed immunohistochemical analysis in the hippocampus (*cornus ammonis* [CA]1, CA3, and dentate gyrus [DG] regions) of *P. gingivalis*-treated obese mice (Figure 4). Representative images of microglia in CA1, CA3, and DG regions of the hippocampus are shown in Figure 4a. The numbers of Iba1-positive cells in the CA3 and DG, but not CA1, regions were significantly increased in the HFD-*Pg* group, compared with the ND-*Pg* group (Figure 4b). The HFD-vehicle group also exhibited a greater number of Iba1-positive cells in the DG, compared with the ND-vehicle group. In the CA3 and DG regions, but not CA1, microglia in the HFD-*Pg* group displayed enlarged cell bodies with an amoeboid shape; the cell area of microglia was significantly greater in the HFD-*Pg* group than in the ND-*Pg* and HFD-vehicle groups. We also analyzed regions of the prefrontal cortex including the prelimbic, infralimbic, and orbitofrontal cortices, which are closely associated with cognition, attention, working memory, and decision making [35] (Figure 5). Representative images of microglia in prelimbic, infralimbic, and orbitofrontal cortices are shown in Figure 5a. We found that the HFD-*Pg* group displayed a significantly increased cell area of microglia in all analyzed regions, compared with the ND-*Pg* and HFD-vehicle groups; however, there were no significant differences in microglial number among groups.

### PLX3397 treatment improved cognitive dysfunction in *P. gingivalis*-treated obese mice

**Figure 6.**
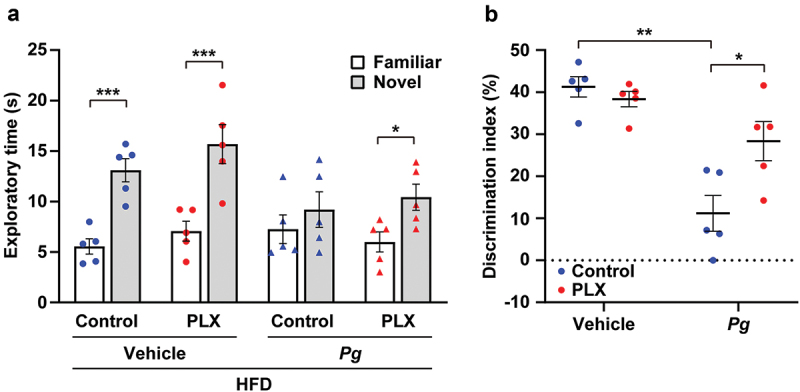
Effects of PLX3397 on cognitive impairment in *P. gingivalis*-treated obese mice. To deplete microglia, PLX3397 (PLX), a CSF1 receptor inhibitor, was orally administered to 35-week-old mice for 10 days. The AIN-76A rodent diet was used as a control (for more details; *see* Materials and Methods). Recognition memory was analyzed using the novel object recognition test. Time spent exploring each object (a) and discrimination index (b) are shown. HFD; high-fat diet, *Pg; P. gingivalis*. Results are expressed as the mean ±S.E.M. of five mice per group. *P < 0.05, **P < 0.01, ***P < 0.001.

To determine whether microglial responses contributed to *P. gingivalis*-induced cognitive dysfunction in obese mice, we depleted microglia in the HFD-*Pg* and HFD-vehicle group by *ad libitum* provision of the CSF1 receptor inhibitor PLX3397 (Figure 6). PLX3397 treatment restored a preference for the novel object in the HFD-*Pg* group; thus, the discrimination index was significantly higher in the PLX3397-treated HFD-*Pg* group than in the control diet-treated HFD-*Pg* group.

## Discussion

In this study, we demonstrated that oral administration of *P. gingivalis* impaired cognitive function in HFD-induced obese mice, through a mechanism involving brain inflammation; moreover, we found that microglia mediated this cognitive dysfunction. A previous study showed that, in middle-aged mice, chronic (5 weeks) systemic exposure to *P. gingivalis* LPS induced an AD-like phenotype, including microglia-mediated neuroinflammation, intracellular amyloid β accumulation in neurons, and learning and memory impairments [36]. A single instance of exposure to *P. gingivalis* LPS activated microglia and astrocytes in the cortex and hippocampus, leading to cognitive impairment [18]. Additionally, ligature-induced acute periodontitis in the molar region causes neuroinflammation and cognitive decline through the STAT3 signaling pathway [37], and ligature-induced periodontal disease-associated microbiome increases activation of microglial cells and enhances phagocytosis of amyloid β via the TLR2/TLR9-mediated pathway [38]. In a more clinically relevant model of periodontal disease, the repeated oral administration of *P. gingivalis* for 22 weeks resulted in neuroinflammation, neurodegeneration, and the formation of intra- and extracellular amyloid plaques and neurofibrillary tangles – pathognomonic signs of AD [39]. Furthermore, repeated oral administration of *P. gingivalis* to AD model mice for 6 weeks aggravated C1q-mediated microglial activation and synaptic pruning and accelerated amyloid β accumulation [40]. Although the association between periodontal pathogens other than *P. gingivalis* and AD has been little studied, *Fusobacterium nucleatum* has been reported to cause microglial activation and cognitive dysfunction [41]. In the present study, we conducted oral administration of *P. gingivalis* and found that even short-term (6 weeks) infection with *P. gingivalis* caused microglial proliferation and activation, along with cognitive impairment, only in HFD-induced obese mice. Periodontal infection had little effect on obesity parameters such as serum leptin levels and liver weight. Although serum *P. gingivalis* antibody titers were comparable between HFD and ND groups, HFD-fed mice with periodontal infection had a significantly lower alveolar crest position, compared with ND-fed non-infected control mice. These findings suggest that obesity can accelerate *P*. gingivalis-induced inflammation and imply that obesity is associated with worsening brain function during periodontal disease progression.

Obesity, a serious global health problem, is linked to multiple diseases, including heart disease, cancer, depression, and neurodegenerative disorders [42–44]. Excessive consumption of an HFD can lead to obesity, metabolic syndrome, and insulin resistance; it may also cause oxidative stress, neuroinflammation, and microglial activation in the brain [14,45–49]. In the present study, we found that the HFD itself increased the number of microglia in the hippocampus, but it did not alter microglial morphology or affect cytokine expression. We suspect that this discrepancy (with respect to previous studies) is related to differences in experimental conditions, such as feeding period, HFD components, and mouse strains. We observed elevated mRNA expression levels of Iba1 and inflammatory cytokines (e.g. IL-1β and TNF-α) in the hippocampus of *P. gingivalis*-treated obese mice. Immunohistochemical analysis also revealed that these mice exhibited increased microglial proliferation and/or morphological changes (i.e. enlarged cell bodies) in the hippocampus and prefrontal cortex. Microglia serve as immunocompetent cells in the central nervous system; they undergo substantial morphological changes in pathological conditions such as neurodegenerative diseases, multiple sclerosis, cerebral infarction, and viral infections, transitioning into an amoeboid shape that is characteristic of activated microglia [50]. These amoeboid microglia have enhanced proliferative, migratory, and phagocytic capabilities; they phagocytose dead cells and eliminate them from lesions. Furthermore, they release bioactive substances such as cytokines, chemokines, and growth factors that are protective to neurons and tissues; they also produce inflammatory factors including reactive oxygen species and inflammatory cytokines that promote damage [51]. Notably, in the present study, the global depletion of microglia by the CSF1 receptor inhibitor PLX3397 ameliorated cognitive dysfunction in *P. gingivalis*-treated obese mice, as determined by the novel object recognition test. These results indicate that microglia play a critical role in regulating cognition during oral *P. gingivalis* infection in the context of obesity, although it remains unknown how microglia affect cognitive function. Microglia play important roles in the regulation of synaptic pruning, neuronal activity, and synaptic plasticity [52–54]. Thus, microglial activation can lead to deficits in synaptic plasticity, learning, and memory [14,26,46]. The novel object recognition test is an experimental behavioral task commonly used to study learning and memory [55]; it is also regarded as a reliable model to evaluate hippocampal and temporal lobe function because lesions within these brain regions disrupt recognition memory [56,57]. Overall, our findings suggest that hippocampal microglia are at least partly involved in the onset of cognitive dysfunction in *P. gingivalis*-treated obese mice.

Thus far, there remains a lack of clarity regarding the mechanism by which oral *P. gingivalis* infection in obesity causes inflammation and microglial activation. Diet-induced obesity reduces blood–brain barrier [58] and intestinal barrier [59] functions. Therefore, components of *P. gingivalis* and pathogen-derived toxins from periodontal tissue may enter the bloodstream and travel to the brain through the blood–brain barrier, leading to microglial activation. In the present study, we observed elevated mRNA expression levels of TLR2 in the hippocampus of *P. gingivalis*-treated obese mice. TLRs have been identified as pattern recognition receptors for pathogen-associated molecular patterns and mediate inflammation and immune responses upon infection [60]. In bacterial and viral central nervous system infections, the participation of receptors TLR2 and TLR4 has been documented [61,62]. Both trigger intracellular signaling pathways with the subsequent increase in the expression of proinflammatory cytokines such as IL-1β and TNF-α [61,63,64]. Additionally, microglia can be activated by various extracellular stimuli, many of which are mediated by TLRs [65,66]. Further research is needed to determine how periodontal disease in the context of diet-induced obesity causes microglial activation.

In conclusion, this study demonstrated that oral administration of *P. gingivalis* in the context of diet-induced obesity triggers an inflammatory response and microglial activation in the brain, leading to impaired cognitive function. These findings will help to clarify the interactive effects of underlying factors on cognitive dysfunction in dementia, which occurs in individuals with many underlying risk factors. The findings will also contribute to the establishment of preventive and therapeutic strategies for cognitive dysfunction based on new molecular pathological information.

## Data Availability

Data will be made available on request.

## References

[1] Nichols E, Steinmetz JD, Vollset SE, et al. Estimation of the global prevalence of dementia in 2019 and forecasted prevalence in 2050: an analysis for the Global Burden of Disease Study 2019. Lancet Public Health. 2022;7(2):e105–13. 34998485 10.1016/S2468-2667(21)00249-8 PMC8810394

[2] Livingston G, Huntley J, Sommerlad A, et al. Dementia prevention, intervention, and care: 2020 report of the Lancet Commission. Lancet. 2020;396(10248):413–446. 32738937 10.1016/S0140-6736(20)30367-6 PMC7392084

[3] Balin BJ, Gérard HC, Arking EJ, et al. Identification and localization of Chlamydia pneumoniae in the Alzheimer’s brain. Med Microbiol Immunol. 1998;187(1):23–42. 9749980 10.1007/s004300050071

[4] Miklossy J, Kis A, Radenovic A, et al. Beta-amyloid deposition and Alzheimer’s type changes induced by Borrelia spirochetes. Neurobiol Aging. 2006;27(2):228–236. 15894409 10.1016/j.neurobiolaging.2005.01.018

[5] Dunn N, Mullee M, Perry VH, et al. Association between dementia and infectious disease: evidence from a case-control study. Alzheimer Dis Assoc Disord. 2005;19(2):91–94. 15942327 10.1097/01.wad.0000165511.52746.1f

[6] Kamer AR, Craig RG, Dasanayake AP, et al. Inflammation and Alzheimer’s disease: possible role of periodontal diseases. Alzheimers Dement. 2008;4(4):242–250. 18631974 10.1016/j.jalz.2007.08.004

[7] Harding A, Singhrao SK. Periodontitis and dementia: a bidirectional relationship? J Dent Res. 2022;101(3):245–246. 34657515 10.1177/00220345211043461 PMC8864330

[8] Hajishengallis G, Liang S, Payne MA, et al. Low-abundance biofilm species orchestrates inflammatory periodontal disease through the commensal microbiota and complement. Cell Host Microbe. 2011;10(5):497–506. 22036469 10.1016/j.chom.2011.10.006 PMC3221781

[9] Maekawa T, Krauss JL, Abe T, et al. Porphyromonas gingivalis manipulates complement and TLR signaling to uncouple bacterial clearance from inflammation and promote dysbiosis. Cell Host Microbe. 2014;15(6):768–778. 24922578 10.1016/j.chom.2014.05.012 PMC4071223

[10] Poole S, Singhrao SK, Kesavalu L, et al. Determining the presence of periodontopathic virulence factors in short-term postmortem Alzheimer’s disease brain tissue. J Alzheimers Dis. 2013;36(4):665–677. 23666172 10.3233/JAD-121918

[11] Dominy SS, Lynch C, Ermini F, et al. Porphyromonas gingivalis in Alzheimer’s disease brains: evidence for disease causation and treatment with small-molecule inhibitors. Sci Adv. 2019;5(1):eaau3333. 30746447 10.1126/sciadv.aau3333 PMC6357742

[12] Olsen I, Singhrao SK. Interaction between genetic factors, Porphyromonas gingivalis and microglia to promote Alzheimer’s disease. J Oral Microbiol. 2020;12(1):1820834. 33062201 10.1080/20002297.2020.1820834 PMC7534375

[13] Kiliaan AJ, Arnoldussen IAC, Gustafson DR. Adipokines: a link between obesity and dementia? Lancet Neurol. 2014;13(9):913–923. 25142458 10.1016/S1474-4422(14)70085-7 PMC4228955

[14] Cope EC, LaMarca EA, Monari PK, et al. Microglia play an active role in obesity-associated cognitive decline. J Neurosci. 2018;38(41):8889–8904. 30201764 10.1523/JNEUROSCI.0789-18.2018 PMC6181311

[15] Yamashita A, Soga Y, Iwamoto Y, et al. Macrophage-adipocyte interaction: marked interleukin-6 production by lipopolysaccharide. Obesity (Silver Spring). 2007;15(11):2549–2552. 18070744 10.1038/oby.2007.305

[16] Nakahara T, Hyogo H, Ono A, et al. Involvement of Porphyromonas gingivalis in the progression of non-alcoholic fatty liver disease. J Gastroenterol. 2018;53(2):269–280. 28741270 10.1007/s00535-017-1368-4

[17] Furusho H, Miyauchi M, Hyogo H, et al. Dental infection of Porphyromonas gingivalis exacerbates high fat diet-induced steatohepatitis in mice. J Gastroenterol. 2013;48(11):1259–1270. 23307045 10.1007/s00535-012-0738-1

[18] Zhang J, Yu C, Zhang X, et al. Porphyromonas gingivalis lipopolysaccharide induces cognitive dysfunction, mediated by neuronal inflammation via activation of the TLR4 signaling pathway in C57BL/6 mice. J Neuroinflammation. 2018;15(1):37. 29426327 10.1186/s12974-017-1052-x PMC5810193

[19] Yamawaki Y, So H, Oue K, et al. Imipramine prevents Porphyromonas gingivalis lipopolysaccharide-induced microglial neurotoxicity. Biochem Biophys Res Commun. 2022;634:92–99. 36240654 10.1016/j.bbrc.2022.09.109

[20] Kilkenny C, Browne W, Cuthill IC, et al. Animal research: reporting in vivo experiments: the ARRIVE guidelines. Br J Pharmacol. 2010;160(7):1577–1579. 20649561 10.1111/j.1476-5381.2010.00872.x PMC2936830

[21] McGrath JC, Lilley E. Implementing guidelines on reporting research using animals (ARRIVE etc.): new requirements for publication in BJP. Br J Pharmacol. 2015;172(13):3189–3193. 25964986 10.1111/bph.12955 PMC4500358

[22] Hamamoto Y, Ouhara K, Munenaga S, et al. Effect of Porphyromonas gingivalis infection on gut dysbiosis and resultant arthritis exacerbation in mouse model. Arthritis Res Ther. 2020;22(1):249. 33076980 10.1186/s13075-020-02348-z PMC7574451

[23] Elmore MR, Najafi AR, Koike MA, et al. Colony-stimulating factor 1 receptor signaling is necessary for microglia viability, unmasking a microglia progenitor cell in the adult brain. Neuron. 2014;82(2):380–397. 24742461 10.1016/j.neuron.2014.02.040 PMC4161285

[24] Mwbjp A. Recognition memory: what are the roles of the perirhinal cortex and hippocampus? Nat Rev Neurosci. 2001;2:51–61. 11253359 10.1038/35049064

[25] Bevins RA, Besheer J. Object recognition in rats and mice: a one-trial non-matching-to-sample learning task to study ‘recognition memory’. Nat Protoc. 2006;1(3):1306–1311. 17406415 10.1038/nprot.2006.205

[26] Hisaoka-Nakashima K, Ohata K, Yoshimoto N, et al. High-mobility group box 1-mediated hippocampal microglial activation induces cognitive impairment in mice with neuropathic pain. Exp Neurol. 2022;355:114146. 35738416 10.1016/j.expneurol.2022.114146

[27] Kawai T, Paster BJ, Komatsuzawa H, et al. Cross-reactive adaptive immune response to oral commensal bacteria results in an induction of receptor activator of nuclear factor-kappaB ligand (RANKL)-dependent periodontal bone resorption in a mouse model. Oral Microbiol Immunol. 2007;22(3):208–215. 17488448 10.1111/j.1399-302X.2007.00348.x

[28] Oue K, Zhang J, Harada-Hada K, et al. Phospholipase C-related catalytically inactive protein is a new modulator of thermogenesis promoted by β-adrenergic receptors in brown adipocytes. J Biol Chem. 2016;291(8):4185–4196. 26706316 10.1074/jbc.M115.705723 PMC4759193

[29] Imado E, Sun S, Abawa AR, et al. Prenatal exposure to valproic acid causes allodynia associated with spinal microglial activation. Neurochem Int. 2022;160:105415. 36027995 10.1016/j.neuint.2022.105415

[30] Yu F, Wang Z, Zhang T, et al. Deficiency of intestinal Bmal1 prevents obesity induced by high-fat feeding. Nat Commun. 2021;12(1):5323. 34493722 10.1038/s41467-021-25674-5 PMC8423749

[31] Napimoga MH, Clemente-Napimoga JT, Macedo CG, et al. Quercetin inhibits inflammatory bone resorption in a mouse periodontitis model. J Nat Prod. 2013;76(12):2316–2321. 24246038 10.1021/np400691n

[32] Munenaga S, Ouhara K, Hamamoto Y, et al. The involvement of C5a in the progression of experimental arthritis with Porphyromonas gingivalis infection in SKG mice. Arthritis Res Ther. 2018;20(1):247. 30390695 10.1186/s13075-018-1744-3 PMC6235227

[33] Jin S, Kim KK, Park BS, et al. Function of astrocyte MyD88 in high-fat-diet-induced hypothalamic inflammation. J Neuroinflammation. 2020;17(1):195. 32560726 10.1186/s12974-020-01846-w PMC7304177

[34] Bettio LEB, Rajendran L, Gil-Mohapel J. The effects of aging in the hippocampus and cognitive decline. Neurosci Biobehav Rev. 2017;79:66–86. 28476525 10.1016/j.neubiorev.2017.04.030

[35] Jobson DD, Hase Y, Clarkson AN, et al. The role of the medial prefrontal cortex in cognition, ageing and dementia. Brain Commun. 2021;3(3):fcab125. 34222873 10.1093/braincomms/fcab125 PMC8249104

[36] Wu Z, Ni J, Liu Y, et al. Cathepsin B plays a critical role in inducing Alzheimer’s disease-like phenotypes following chronic systemic exposure to lipopolysaccharide from Porphyromonas gingivalis in mice. Brain Behav Immun. 2017;65:350–361. 28610747 10.1016/j.bbi.2017.06.002

[37] Hu Y, Zhang X, Zhang J, et al. Activated STAT3 signaling pathway by ligature-induced periodontitis could contribute to neuroinflammation and cognitive impairment in rats. J Neuroinflammation. 2021;18(1):80. 33757547 10.1186/s12974-021-02071-9 PMC7986277

[38] Almarhoumi R, Alvarez C, Harris T, et al. Microglial cell response to experimental periodontal disease. J Neuroinflammation. 2023;20(1):142. 37316834 10.1186/s12974-023-02821-x PMC10265806

[39] Ilievski V, Zuchowska PK, Green SJ, et al. Chronic oral application of a periodontal pathogen results in brain inflammation, neurodegeneration and amyloid beta production in wild type mice. PLOS ONE. 2018;13(10):e0204941. 30281647 10.1371/journal.pone.0204941 PMC6169940

[40] Hao X, Li Z, Li W, et al. Periodontal infection aggravates C1q-mediated microglial activation and synapse pruning in Alzheimer’s Mice. Front Immunol. 2022;13:816640. 35178049 10.3389/fimmu.2022.816640 PMC8845011

[41] Wu H, Qiu W, Zhu X, et al. The periodontal pathogen fusobacterium nucleatum exacerbates Alzheimer’s pathogenesis via specific pathways. Front Aging Neurosci. 2022;14:912709. 35813949 10.3389/fnagi.2022.912709 PMC9260256

[42] Yau PL, Castro MG, Tagani A, et al. Obesity and metabolic syndrome and functional and structural brain impairments in adolescence. Pediatrics. 2012;130(4):e856–864. 22945407 10.1542/peds.2012-0324 PMC3457620

[43] Morris MJ, Beilharz JE, Maniam J, et al. Why is obesity such a problem in the 21st century? The intersection of palatable food, cues and reward pathways, stress, and cognition. Neurosci Biobehav Rev. 2015;58:36–45. 25496905 10.1016/j.neubiorev.2014.12.002

[44] Pedditzi E, Peters R, Beckett N. The risk of overweight/obesity in mid-life and late life for the development of dementia: a systematic review and meta-analysis of longitudinal studies. Age Ageing. 2016;45(1):14–21. 26764391 10.1093/ageing/afv151

[45] Bruce-Keller AJ, White CL, Gupta S, et al. NOX activity in brain aging: exacerbation by high fat diet. Free Radic Biol Med. 2010;49(1):22–30. 20347034 10.1016/j.freeradbiomed.2010.03.006 PMC2875353

[46] Hao S, Dey A, Yu X, et al. Dietary obesity reversibly induces synaptic stripping by microglia and impairs hippocampal plasticity. Brain Behav Immun. 2016;51:230–239. 26336035 10.1016/j.bbi.2015.08.023 PMC4679537

[47] Spencer SJ, D’Angelo H, Soch A, et al. High-fat diet and aging interact to produce neuroinflammation and impair hippocampal- and amygdalar-dependent memory. Neurobiol Aging. 2017;58:88–101. 28719855 10.1016/j.neurobiolaging.2017.06.014 PMC5581696

[48] Jeong MY, Jang HM, Kim DH. High-fat diet causes psychiatric disorders in mice by increasing Proteobacteria population. Neurosci Lett. 2019;698:51–57. 30615977 10.1016/j.neulet.2019.01.006

[49] Guo DH, Yamamoto M, Hernandez CM, et al. Visceral adipose NLRP3 impairs cognition in obesity via IL-1R1 on CX3CR1+ cells. J Clin Invest. 2020;130(4):1961–1976. 31935195 10.1172/JCI126078 PMC7108893

[50] Walker FR, Nilsson M, Jones K. Acute and chronic stress-induced disturbances of microglial plasticity, phenotype and function. Curr Drug Targets. 2013;14(11):1262–1276. 24020974 10.2174/13894501113149990208 PMC3788324

[51] Hanisch UK. Microglia as a source and target of cytokines. Glia. 2002;40(2):140–155. 12379902 10.1002/glia.10161

[52] Cornell J, Salinas S, Huang HY, et al. Microglia regulation of synaptic plasticity and learning and memory. Neural Regen Res. 2022;17(4):705–716. 34472455 10.4103/1673-5374.322423 PMC8530121

[53] De Felice E, de Andrade E G, Golia MT, et al. Microglial diversity along the hippocampal longitudinal axis impacts synaptic plasticity in adult male mice under homeostatic conditions. J Neuroinflammation. 2022;19(1):292. 36482444 10.1186/s12974-022-02655-z PMC9730634

[54] Hikosaka M, Kawano T, Wada Y, et al. Immune-triggered forms of plasticity across brain regions. Front Cell Neurosci. 2022;16:925493. 35978857 10.3389/fncel.2022.925493 PMC9376917

[55] Ennaceur A. One-trial object recognition in rats and mice: methodological and theoretical issues. Behav Brain Res. 2010;215(2):244–254. 20060020 10.1016/j.bbr.2009.12.036

[56] Winters BD, Saksida LM, Bussey TJ. Object recognition memory: neurobiological mechanisms of encoding, consolidation and retrieval. Neurosci Biobehav Rev. 2008;32(5):1055–1070. 18499253 10.1016/j.neubiorev.2008.04.004

[57] Broadbent NJ, Gaskin S, Squire LR, et al. Object recognition memory and the rodent hippocampus. Learn Mem. 2010;17(1):5–11. 20028732 10.1101/lm.1650110 PMC2807177

[58] Yamamoto M, Guo DH, Hernandez CM, et al. Endothelial Adora2a activation promotes blood-brain barrier breakdown and cognitive impairment in mice with diet-induced insulin resistance. J Neurosci. 2019;39(21):4179–4192. 30886019 10.1523/JNEUROSCI.2506-18.2019 PMC6529868

[59] Zhang P, Yu Y, Qin Y, et al. Alterations to the microbiota-colon-brain axis in high-fat-diet-induced obese mice compared to diet-resistant mice. J Nutr Biochem. 2019;65:54–65. 30623851 10.1016/j.jnutbio.2018.08.016

[60] Chopra A, Bhat SG, Sivaraman K. Porphyromonas gingivalis adopts intricate and unique molecular mechanisms to survive and persist within the host: a critical update. J Oral Microbiol. 2020;12(1):1801090. 32944155 10.1080/20002297.2020.1801090 PMC7482874

[61] Lehnardt S. Innate immunity and neuroinflammation in the CNS: the role of microglia in Toll-like receptor-mediated neuronal injury. Glia. 2010;58(3):253–263. 19705460 10.1002/glia.20928

[62] Olson JK, Miller SD. Microglia initiate central nervous system innate and adaptive immune responses through multiple TLRs. J Immunol. 2004;173(6):3916–3924. 15356140 10.4049/jimmunol.173.6.3916

[63] Kawai T, Akira S. TLR signaling. Cell Death Differ. 2006;13(5):816–825. 16410796 10.1038/sj.cdd.4401850

[64] Esen N, Kielian T. Central role for MyD88 in the responses of microglia to pathogen-associated molecular patterns. J Immunol. 2006;176(11):6802–6811. 16709840 10.4049/jimmunol.176.11.6802 PMC2440502

[65] Rivest S. Regulation of innate immune responses in the brain. Nat Rev Immunol. 2009;9(6):429–439. 19461673 10.1038/nri2565

[66] Nie X, Kitaoka S, Tanaka K, et al. The innate immune receptors TLR2/4 mediate repeated social defeat stress-induced social avoidance through prefrontal microglial activation. Neuron. 2018;99(3):464–479.e467. 30033154 10.1016/j.neuron.2018.06.035

